# Detectability of cerebellar activity with magnetoencephalography and electroencephalography

**DOI:** 10.1002/hbm.24951

**Published:** 2020-03-01

**Authors:** John G. Samuelsson, Padmavathi Sundaram, Sheraz Khan, Martin I. Sereno, Matti S. Hämäläinen

**Affiliations:** ^1^ Harvard‐MIT Division of Health Sciences and Technology (HST) Massachusetts Institute of Technology (MIT) Cambridge Massachusetts USA; ^2^ Athinoula A. Martinos Center for Biomedical Imaging Massachusetts General Hospital Charlestown Massachusetts USA; ^3^ Harvard Medical School Boston Massachusetts USA; ^4^ Department of Psychology and Neuroimaging Center San Diego State University San Diego California USA; ^5^ Experimental Psychology University College London London UK

**Keywords:** cerebellum, EEG, forward modeling, MEG, Monte Carlo simulations, signal cancellation

## Abstract

Electrophysiological signals from the cerebellum have traditionally been viewed as inaccessible to magnetoencephalography (MEG) and electroencephalography (EEG). Here, we challenge this position by investigating the ability of MEG and EEG to detect cerebellar activity using a model that employs a high‐resolution tessellation of the cerebellar cortex. The tessellation was constructed from repetitive high‐field (9.4T) structural magnetic resonance imaging (MRI) of an ex vivo human cerebellum. A boundary‐element forward model was then used to simulate the M/EEG signals resulting from neural activity in the cerebellar cortex. Despite significant signal cancelation due to the highly convoluted cerebellar cortex, we found that the cerebellar signal was on average only 30–60% weaker than the cortical signal. We also made detailed M/EEG sensitivity maps and found that MEG and EEG have highly complementary sensitivity distributions over the cerebellar cortex. Based on previous fMRI studies combined with our M/EEG sensitivity maps, we discuss experimental paradigms that are likely to offer high M/EEG sensitivity to cerebellar activity. Taken together, these results show that cerebellar activity should be clearly detectable by current M/EEG systems with an appropriate experimental setup.

## 1 INTRODUCTION

The cerebellum contains more than 70% of all neurons in the human brain and is heavily interconnected with the cerebral cortex (Andersen, Korbo, & Pakkenberg, 1992; Herculano‐Houzel, 2009). The literature over the past three decades has provided evidence for cerebellar involvement in some of the most prevalent neurological diseases ranging from schizophrenia (Cengiz & Boran, 2016; Picard, Amado, Mouchet‐Mages, Olié, & Krebs, 2007) to essential tremor (Cerasa & Quattrone, 2016; Choe et al., 2016; Filip, Lungu, Manto, & Bareš, 2016; Gironell, 2014; Grimaldi & Manto, 2013; Handforth, 2016; Schnitzler, Münks, Butz, Timmermann, & Gross, 2009), and Parkinson's disease (Ma, Tang, Spetsieris, Dhawan, & Eidelberg, 2007; Timmermann et al., 2004; Wu et al., 2009; Wu & Hallett, 2013; Yu, Sternad, Corcos, & Vaillancourt, 2007). Despite its central role in executive function, motor control and cognition, as well as involvement in some of the most common neurological disorders, the cerebellum has remained largely overlooked in both basic neuroscience and clinical research (Schmahmann & Sherman, 1998a; Wu & Hallett, 2013). Although positron emission tomography and functional magnetic resonance imaging (MRI) have been valuable tools in assessing cerebellar metabolism and regional blood flow (Stoodley & Schmahmann, 2009), cerebellar electrophysiology in humans remains poorly characterized (Dalal, Osipova, Bertrand, & Jerbi, 2013). Electrophysiological techniques such as magnetoencephalography (MEG) and electroencephalography (EEG) that directly detect neuronal activity noninvasively at a high temporal resolution are required to fill this gap.

However, noninvasive detection of cerebellar electrophysiology using M/EEG has traditionally been assumed to be challenging because of the remote location of the cerebellum and its finely convoluted cortex. The literature shows a lack of studies that attempt to validate or refute this position, one reason being the lack of a detailed model to simulate the M/EEG signals resulting from neural activity in the cerebellar cortex. Because we require such a model in order to assess detectability and source mapping of cerebellar activity with M/EEG, there has been no quantification of the expected signal strength and cancelation effects, nor detailed M/EEG sensitivity maps of the cerebellum. These analytical results will be useful in assessing detectability of cerebellar activity, interpreting M/EEG data, and in designing suitable future studies to acquire such data.

The literature of studies on cerebellar electrophysiology is relatively sparse. Few EEG studies of the human cerebellum have been conducted and they have mainly focused on cerebellar activity during saccades, epileptic discharges, or motor learning (Lascano et al., 2013; Mehrkanoon, Boonstra, Breakspear, Hinder, & Summers, 2016; Todd, Govender, & Colebatch, 2018). Other studies have recorded EEG of patients with cerebellar lesions and have reported event‐related potentials (ERP) changes but without any source estimation (Peterburs et al., 2013a, 2013b; Peterburs et al., 2015). This lack of cerebellar source estimates may be because cerebellar EEG is assumed to contain significant myogenic contaminations (Muthukumaraswamy, 2013). In comparison to EEG, the literature of MEG studies is somewhat larger. Reported MEG studies have elicited cerebellar activity via saccades and conditioned eye blinks (Ioannides & Fenwick, 2005; Ioannides, Fenwick, & Liu, 2005; Jousmaki, Hamalainen, & Hari, 1996; Kirsch et al., 2003, Lin et al., 2019), median nerve stimulation (Hashimoto, Kimura, Tanosaki, Iguchi, & Sekihara, 2003; Ioannides & Fenwick, 2005; Tesche & Karhu, 1997), finger tapping (Marty et al., 2018; Muthuraman et al., 2014) and presentation of faces with emotional expressions (Ioannides, Poghosyan, Dammers, & Streit, 2004). Reported invasive recordings in the human cerebellum are scarce; Niedermeyer (2004) gives an overview of the more commonly known cases. In Dalal et al. (2013), the authors review lesser known cases of intracranial electrocerebellogram in humans and survey MEG and EEG studies that report cerebellar activity. Andersen, Jerbi, and Dalal (2019) did an extensive review of M/EEG studies that report cerebellar activity and also present methodological suggestions for increasing the detectability of cerebellar signals. Although these studies shed promising light upon the investigation of cerebellar electrophysiology recorded with MEG, the cerebellar activity itself was not the primary focus in many of these studies and was often reported as a secondary finding. One reason for the relatively small body of literature in this subject area is the prevailing uncertainty about the feasibility of recording cerebellar electrophysiology with MEG and EEG. In this study, we aim to bridge this gap by quantifying the detectability of cerebellar activity with MEG and EEG.

To compute the signals produced by cerebellar currents, a geometrical model of the brain structure is required since the location and orientation of the neural current sources are needed for simulating the resulting electric potentials and magnetic fields on the scalp. An accurate geometric model of the cerebellar cortex has so far been unattainable due to conventional MRI's inability to resolve the folia of the cerebellum at sufficient resolution. Since the human cerebellum is more tightly folded than the neocortex with individual folia being 1–2 mm wide (Braitenberg & Atwood, 1958) and the complete cerebellar cortical sheet approximately 1.5–2 m long (Sultan & Braitenberg, 1993), we would require an MRI image resolution of less than 200 μm to resolve the folds (Sereno, Diedrichsen, Tachrount, Silva, & De Zeeuw, 2014); this is not possible with the typical in vivo MRI image resolution of 2–4 mm. In this study, therefore, we used a tessellation of the cerebellar cortical surface based on repetitive high‐field (9.4T) MRI scans of an ex vivo human cerebellum; since these MRI data had an image resolution of 0.19 x 0.19 x 0.19 mm^3^, the geometry of the cerebellar cortex could be resolved accurately (Sereno, Diedrichsen, Tachrount, Silva, & Zeeuw, 2015). This detailed geometrical model of the cerebellum was then added to the source space of a healthy subject with the cerebral source space based on conventional MRI. The current density of the neural sources underlying the M/EEG signals was assigned using the work of Murakami and Okada (2015); the authors found that the current dipole moment density in neural tissue is largely invariant (≈1 nAm/mm^2^) across brain structures such as neocortex, hippocampus, and the cerebellum, as well as across species, ranging from turtle to human. Using this result as a physiological constraint, we were able to model neural activity in the cerebellum in a similar way to that typically done in the cerebral cortex. We then computed the resulting MEG and EEG signals using established methods (Hämäläinen, Hari, Ilmoniemi, Knuutila, & Lounasmaa, 1993; Hämäläinen & Sarvas, 1989; Mosher, Leahy, & Lewis, 1999).

The rest of the article is organized as follows. In Section 2, we describe construction of the cerebellar source space using a high‐resolution tessellation obtained from high‐field MRI of an ex vivo human cerebellum as well as using a lower resolution surface obtained from the FreeSurfer software package for comparison (Fischl, 2012); we then describe how the cerebellar source space was combined with the cortical source space to perform forward calculations that estimate MEG and EEG signals. We then define how we quantified the signal cancelation due to unaligned neural current sources, using measures related to those presented in Ahlfors et al. (2010) for the cortex; we considered both randomly distributed dipole sources as well as spatially coherent patches. In Section 3, we present the results of our simulations using the high‐resolution model of the cerebellum and compare to results using the FreeSurfer model of the cerebellum and the FreeSurfer reconstruction of the cortex. We compare MEG and EEG signal cancelation and signal strength for both distributed and spatially coherent source configurations. We then present detailed sensitivity maps of MEG magnetometers, gradiometers, and EEG, respectively, using the high‐resolution cerebellum model. Finally, we present a discussion of our results and conclusion.

## 2 METHODS

### 2.1 Modeling

#### 2.1.1 The model of the cerebellum

T2*‐weighted (flip angle = 20^o^, TR/TE = 30/18 ms) and proton density (flip angle = 10^o^, TR/TE = 15/3.7 ms) 3D FLASH sequence images of a well‐preserved ex vivo human cerebellum from a 62‐year‐old Caucasian female in Fomblin were obtained with an isotropic voxel resolution of 0.19 x 0.19 x 0.19 mm^3^ at 9.4T (Agilent Technologies, Inc., Santa Clara, CA). Each scan was repeated 10 times (total scan time: 12 hr). An initial reconstruction from the 512 x 340 x 340 voxel volume was repeatedly edited manually and re‐tessellated to remove topological defects and then refined to obtain a triangulated surface near the Purkinje cell layer. The resulting tessellation contained 4,580,006 vertices and 9,176,308 triangular faces with an average edge length of 0.16 mm (160 μm).

#### 2.1.2 Construction of the source space

**Figure 1:**
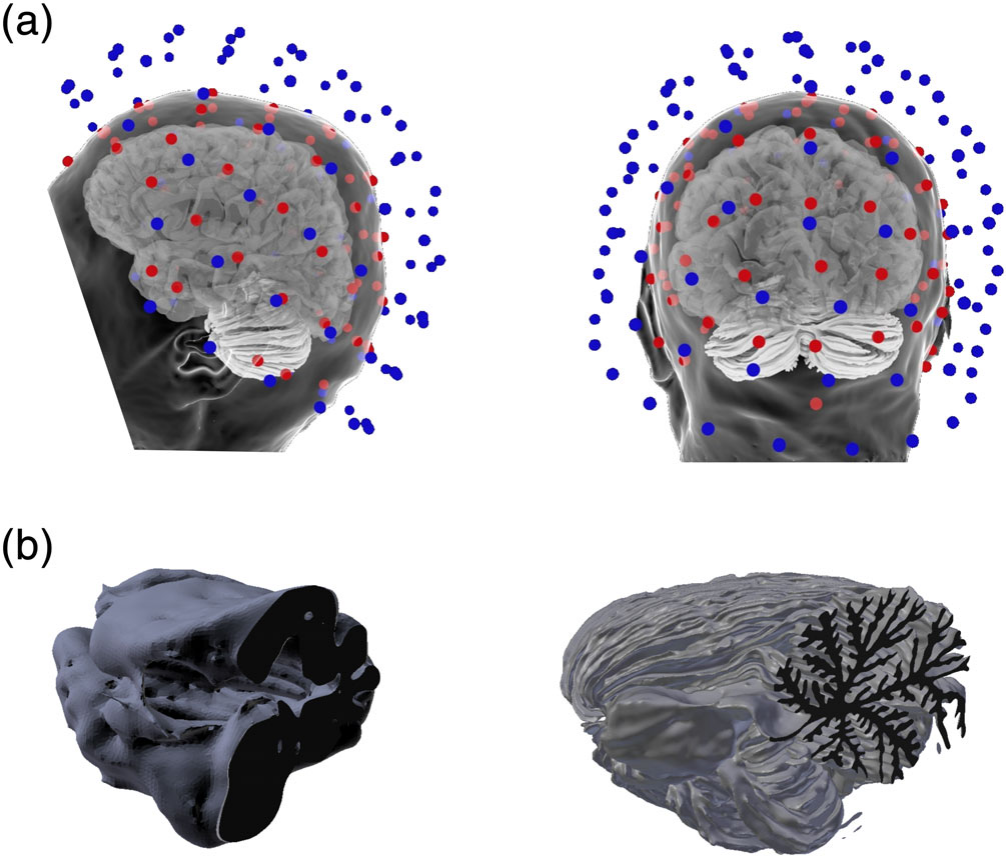
(a) The MEG and EEG sensor arrays with the head surface and cortical and cerebellar source reconstructions. The MEG sensors are colored blue to distinguish them from the EEG sensors (red) and the head surface is translucent to allow for viewing of the cerebellum and cortex in relation to the sensor arrays. (b) Sagittal plane cross‐section of the cerebellar vermis of the standard FreeSurfer tessellation of the cerebellum (left) and the detailed model used in this study (right)

Structural MRI data were collected after informed consent from a healthy 60‐year‐old male under a protocol approved by the Massachusetts General Hospital Institutional Review Board. The subject had no medical history of any neurological disorder. T1‐weighted, high‐resolution Magnetization Prepared Rapid Gradient Echo structural images were acquired on a 1.5T Siemens whole‐body MRI scanner (Siemens Medical Systems, Erlangen, Germany) using a 32‐channel head coil. Using FreeSurfer (Fischl, 2012), the cortical surface was reconstructed and automated segmentation was used to extract an approximate surface of the subject's cerebellum (Figure 1). As the detailed cerebellar surface mesh was obtained from an ex vivo MRI of a different subject, an affine transformation was used to match the location, orientation, and size of the detailed cerebellar model to the coarse FreeSurfer reconstruction, which is an outline of the cerebellum specific to the subject. The transformation was done using trimesh2 software package (Rusinkiewicz, 2004). The cerebellum model was then loaded into MNE‐Python (Gramfort et al., 2013) and combined with the source space consisting of the left and right cerebral hemispheres.

We performed a mesh convergence study to verify that the full mesh of 4,579,483 source points was sufficiently dense to adequately compute the M/EEG signals. Three downsampled versions of the cerebellar cortex were obtained using the implementation of the Dijkstra algorithm (Dijkstra, 1959) in MNE‐Python with 273,919, 504,509, and 2,524,356 vertices, respectively. The sensor space signal difference between the full mesh and the downsampled mesh with 2,524,356 vertices was consistently less than 1%. Based on these results, we concluded that the mesh had converged and that using a mesh denser than the highest resolution mesh with 4,579,483 vertices would have a negligible impact on the results. Figure 1 shows the standard FreeSurfer segmentation of the cerebellum (B, left) and the detailed, full mesh model of the cerebellum used in this study (B, right). Figure 1a shows two views of the location of the cerebellum in the head along with the MEG (blue) and EEG (red) sensor arrays.

#### 2.1.3 The source model

A forward model was constructed after adding the cerebellum model to the source space consisting of a tessellation of the cortical mantle of the cerebral hemispheres. The forward model was used to calculate the M/EEG signal based on neural activity modeled as current dipoles placed at the vertices of the source space and aligned with the vertex normals (fixed source configuration). The vertex normals were calculated as an angle‐weighted sum of normals of the incident faces using the Meshlab software package (Cignoni et al., 2008; Thürrner & Wüthrich, 1998). In the cerebral cortex, current dipoles modeled dendritic currents in the pyramidal neurons as is routine in M/EEG. Correspondingly, in the cerebellar cortex, current dipoles modeled dendritic currents in the Purkinje neurons. These currents have previously been shown to be the physiological source of the M/EEG signals and can be assumed to generate the same current density as the pyramidal dendritic currents in the cerebral cortex (1 nAm/mm^2^; Hämäläinen et al., 1993; Murakami & Okada, 2015; Okada, 1989). Thus, neural current sources in the cerebellum can be modeled in a way similar to that in the neocortex.

#### 2.1.4 MEG/EEG field computations

The simulated scalp magnetic fields and electric potentials were assumed to be measured with a 306‐channel Vectorview MEG system (Elekta‐Neuromag Oy, Helsinki, Finland) and a 72‐channel MEG‐compatible EEG cap (EasyCap GmbH, Herrsching, Germany). The locations of the MEG array and the EEG electrodes were taken from an actual M/EEG measurement of the subject whose MRI data we used for the FreeSurfer reconstruction. The M/EEG and the MRI were registered using the locations of three fiduciary points (nasion and left/right auricular points) that define a head‐based coordinate system, a set of points from the head surface, and the sites of the four HPI coils that were digitized using a Fastrak digitizer (Polhemus) integrated with the VectorView system.

A three‐compartment piecewise homogenous conductor model of the head with conductivities 0.3, 0.006, and 0.3 S/m for brain, skull, and scalp, respectively, and boundary‐element method with linear collocation in the field computations were used for building the forward model (Mosher et al., 1999). All source vertices within 5 mm from the inner skull boundary were excluded to avoid source locations resulting in numerical errors. 127,580 (2.8%) source points in the cerebellum and 251 source points in the cortex (0.08%) were excluded due to this constraint, resulting in a total of 4,451,903 active source points in the cerebellum and 308,354 active source points in the cortex.

### 2.2 Simulations

#### 2.2.1 Signal cancelation: Conservation factor

To quantify the signal cancelation due to unaligned neural current sources, we used measures related to those presented in Ahlfors et al. (2010), that is, the signal generated by *n* simultaneous sources was compared with the sum of the signals generated by the same sources when individually activated.

The net signal in the sensor array generated by *n* simultaneously active unit current dipole sources can be quantified as

$$Α\left(n\right)={\left\|\sum_{j}^{n}G_{:,j}\right\|}_{2},\;{\left\|\sum_{j}^{n}G_{:,j}\right\|}_{2}=\sqrt{\sum_{i=1}^{M}{\left(\sum_{j=1}^{n}g_{\mathrm{ij}}\right)}^{2}},$$

where *M* is the number of sensors, *G*_:, *j*_ is the forward solution for current dipole *j* and *g*_*ij*_ are the elements of the (*M x N*)‐dimensional forward matrix *G*, where *N* is the total number of points in the source space.

When each of the *n* dipoles is active individually, the sum of the resulting signal norms is,

$$Β\left(n\right)=\sum_{j=1}^{n}{\left\|G_{:,j}\right\|}_{2}=\sum_{j=1}^{n}\sqrt{\sum_{i=1}^{M}g_{\mathrm{ij}}^{2}}.$$

The net signal Α is equal to the absolute signal Β times the conservation factor *C*, which quantifies the signal cancelation, *A* = *CB* and hence, *C* = *A*/*B*.

The conservation factor *C* will thus be between 0 and 1. If the net and absolute signals are the same (*Α* = *Β*), there is no cancelation at all and the conservation factor *C* = 1. If *Α* = 0 and *Β* ≠ 0, that is, there is an absolute signal but no net signal, there is complete signal cancelation and *C* = 0. Note that in Ahlfors et al. (2010), the cancelation was quantified as the cancelation index *I*_*c*_ = 1 − *C*.

To ensure that the current dipole density in the activated cortical patches was 1 nAm/mm^2^, the computed signal norms *Α* and *Β* were divided by the number of active dipoles in the patch *n* and then multiplied by the activated area *a*_*n*_ and the scale factor *q*_0_ = 10^−9^Am/mm^2^;

$$\alpha \left(n\right)=\frac{Α}{n}a_{n}q_{0},$$

$$\beta \left(n\right)=\frac{B}{n}a_{n}q_{0}.$$

The current amplitudes are now physiologically correct and the signal norms *α* and *β* are invariant to grid density in the source space mesh. Since *α* and *β* are derived from *Α* and *Β* by the same scale factor, we have *C* = *α*/*β*.

#### 2.2.2 Monte Carlo simulations

Monte Carlo simulations were performed to quantify signal cancelation as *C*, the net signal *α* and the absolute signal *β* in the cortex and the two cerebellum models. We varied the number of activated dipoles *n* and performed the simulations for two source configurations: (1) “distributed dipoles,” where the dipoles were selected randomly from all over the cerebral and cerebellar cortex and (2) “spatially coherent sources,” where the dipoles were placed as spatially coherent patches of uniform activity. For spatially coherent sources, the center of the patch was selected randomly and neighboring vertices were added recursively until the patch attained the desired surface area. Two hundred different activations of both distributed and spatially coherent sources were created for use in the simulations; the coherent sources had radii ranging from 1 to 30 mm while the distributed sources had a varying number of dipoles ranging from *n* = 1 to *n* = *N*, covering the full surface area.

## 3 RESULTS

We will henceforth refer to the cerebral cortex as “cortex,” our high‐resolution cerebellum model as “cerebellum” and the FreeSurfer outer shell segmentation of the cerebellum as “FreeSurfer cerebellum.”

### 3.1 Signal cancelation

#### 3.1.1 Distributed dipoles

**Figure 2:**
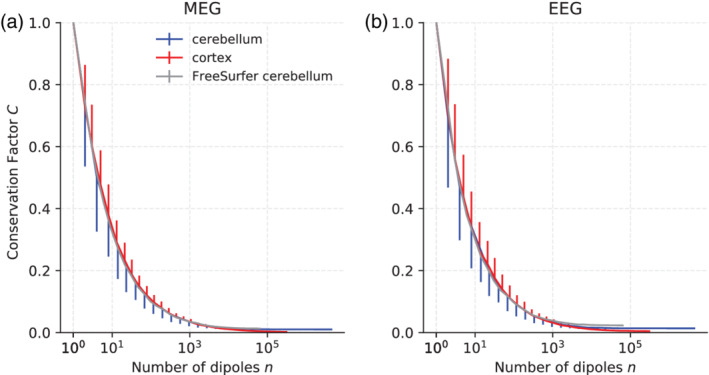
Conservation factor *C* as a function of the number of randomly distributed current dipoles in the cortex (red), the high‐resolution cerebellum model (blue), and the FreeSurfer cerebellum model (gray) for MEG magnetometers (left) and EEG (right). For each *n*, the mean conservation factor is plotted. The bars represent one standard deviation and are plotted one‐sided only for clarity

When the current dipoles were randomly distributed, there was no significant difference in signal cancelation between the cortex, the high‐resolution cerebellum, and the FreeSurfer cerebellum with MEG or EEG (Figure 2). There was also almost no difference in cancelation between MEG magnetometers, gradiometers, and EEG (Figure 2). Because the cancelation in MEG magnetometers and gradiometers were virtually the same, Figure 2 shows only simulation results for magnetometers and EEG. We note the fast decline in conservation factor when very few dipoles were activated; the conservation factor was less than 0.5 for fewer than 10 active dipole sources (Figure 2). For a large number of randomly distributed dipoles (*n* > 10,000 dipoles), the conservation factor converged towards *C* = 0, that is, close to complete cancelation of M/EEG signals. These results show that signal cancelation for distributed sources is significant and of similar magnitude in the cerebellum and cortex with both MEG and EEG.

#### 3.1.2 Spatially coherent dipoles

**Figure 3:**
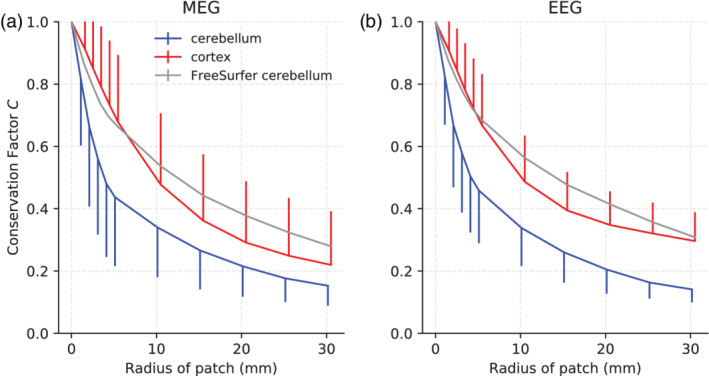
Conservation factor *C* estimated by Monte Carlo simulations resulting from activated cortical patches of varying radii and uniform neural current density in the cortex, cerebellum, and the FreeSurfer cerebellum for (a) MEG magnetometers and (b) EEG. For each patch size, the mean conservation factor is plotted. The bars represent one standard deviation and are plotted one‐sided for clarity

Figure 3 shows the conservation factor as a function of patch radius when spatially coherent dipoles were activated. The conservation factor decreased monotonically with patch size (Figure 3). Just like we observed for distributed sources in Figure 2, signal cancelation for spatially coherent sources was virtually the same in MEG magnetometers as in gradiometers. Figure 3 therefore only shows the results for magnetometers and EEG.

The FreeSurfer cerebellum, which smoothed over the cerebellar folia (Figure 1), resulted in a gross overestimation of the conservation factor. The conservation factor using the FreeSurfer cerebellum model was even greater than the conservation factor of the cortex for patches of radius greater than 5 mm; this was true in both EEG and MEG. This result was likely because the FreeSurfer cerebellar surface was a coarse description of the cerebellar surface with barely any sulci at all, therefore underestimating the signal cancelation due to oppositely oriented dipoles in opposing sulcal walls and consequently overestimating the conservation factor and the net signal. This result highlights the inadequacy of using the FreeSurfer segmentation of the cerebellum as a source model for M/EEG forward calculations. We will therefore only use our high‐resolution cerebellum model in the remainder of this article. The high‐resolution cerebellum model and the FreeSurfer cortex reconstruction are therefore referred to simply as “cerebellum” and “cortex,” respectively.

The conservation factor in the cerebellum was generally lower than in the cortex, implying a higher degree of signal cancelation. The cerebellar conservation factor was 10–50% lower than the cortical one, depending on patch size and measurement modality. This was expected given the tightly folded cerebellar cortex, which was resolved in our cerebellum model. The conservation factor was higher in EEG than in MEG for cortical signals but about the same in the cerebellum. Particularly characteristic for the cerebellum was the steep fall in conservation factor for relatively small patches, implying significant signal cancelation even for smaller activated areas. The mean conservation factor in the cerebellum was less than 0.5 for patches with radius larger than 4 mm; the conservation factor in the cortex was less than 0.5 for patches of radius larger than 10 mm.

The variation in conservation factor was higher in the cortex than in the cerebellum for larger patches in both MEG and EEG. In MEG, for example, the standard deviation of the conservation factor for patches of radius 30 mm was 0.2 in the cortex and 0.1 in the cerebellum. This result reflects the geometric heterogeneity of the cerebral cortex as compared to the cerebellar cortex.

### 3.2 Signal strength

#### 3.2.1 Individual dipole sources

**Figure 4:**
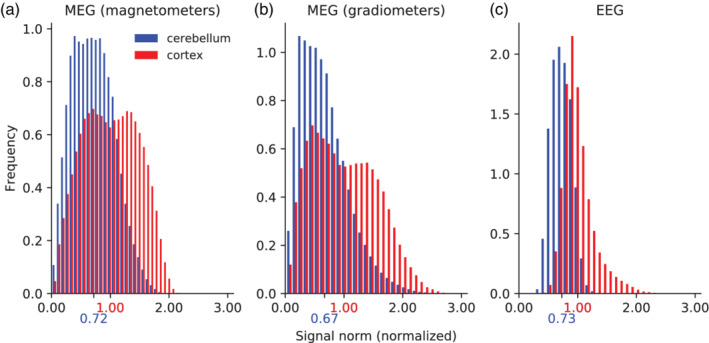
Histogram plots of the signal norm from individually activated dipoles in the cerebellum and cortex for MEG magnetometers (a), MEG gradiometers (b), and EEG (c). The signal norms were normalized to the median cortical signal norm, marked on the *x*‐axis in red. The median cerebellar signal norms are marked on the *x*‐axis in blue. The distributions were normalized so that the integral of each distribution equals one

The M/EEG net signal is not only a function of the cancelation effect but also of the absolute signal, which depends on location and orientation of the dipole sources with respect to the sensors. We therefore calculated the M/EEG signal norms due to individually activated current dipoles in the cerebellum and the cortex; the histograms are shown in Figure 4 and represent all the dipoles in the cerebellar and cortical source spaces, respectively. As the dipoles were activated individually, signal cancelation is not a factor and any differences in signal norm were solely due to location and orientation of the current dipoles in relation to the sensor locations. The signal strength from individual dipoles in the cerebellum and in the cortex were of the same order of magnitude in both MEG and EEG, the mean cerebellar signal norm being about 30% smaller than the mean cortical signal norm in both MEG and EEG. The variation in signal strength was generally larger in MEG than EEG (Figure 4). In approximately 19% of the cases, individual current dipoles in the cerebellum gave a stronger signal than the median signal norm from cortical dipoles across all modalities.

#### 3.2.2 Spatially coherent dipoles

**Figure 5:**
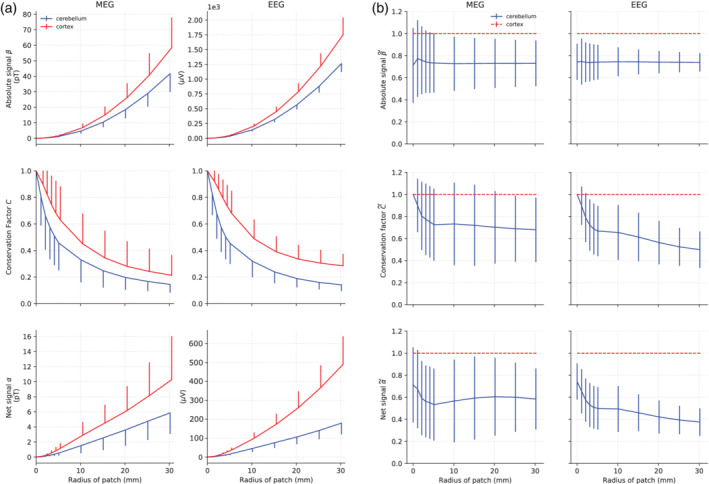
(a) The absolute signal (*β*, top row), the conservation factor (*C*, middle row), and the net signal (*α*, bottom row) as a function of patch radius (*r*) for the cerebellum (blue) and the cortex (red). These are related by the equation *α* = *Cβ*. For each patch size, 200 patch samples at random locations were drawn. (b) The normalized absolute signal ($\tilde{\beta }$, top row), the normalized conservation factor ($\tilde{C}$, middle row) and the normalized net signal ($\tilde{\alpha }$, bottom row) as a function of patch radius (*r*) for the cerebellum (blue). The mean cortical values, which were used as reference to normalize the corresponding cerebellar values, are marked as the dashed red line. In both (a) and (b), the left column is MEG magnetometers and the right column is EEG. The bars represent one standard deviation and are plotted one‐sided for clarity in (a). The data were generated using Monte Carlo simulations

We also computed the net signal norm *α* for the different patches. These are shown in Figure 5a (bottom row) alongside the absolute signal *β* (top row) and the conservation factor *C* (middle row) as a function of patch radius. As in Figure 3, the conservation factor decreased monotonically with patch size, approximately as the inverse of the patch radius. The absolute signal *β* increased approximately linearly with patch area and thus quadratically with patch radius (Figure 5, top row). The resulting net signal *α* therefore increased approximately linearly with patch radius, since *α* = *Cβ*. This means that a greater activation area resulted in a stronger signal, despite the increased cancelation effect; this fact is important when choosing an experimental stimulus to elicit a cerebellar response.

Figure 5b is similar to Figure 5a except that the net signal (*α*), the absolute signal (*β*), and the conservation factor (*C*) are normalized to the average values of the cortex for that patch size. Results for the cerebellum were plotted as a function of patch radius *r* and included the normalized net signal ($\tilde{\alpha }$), normalized absolute signal ($\tilde{\beta }$), and the normalized conservation factor ($\tilde{C}$), given as;

$$\tilde{C}\left(r\right)=\frac{C_{\mathrm{cb}}\left(r\right)}{\bar{C_{\mathrm{ctx}}}\left(r\right)},$$

$$\tilde{\alpha }\left(r\right)=\frac{\alpha_{\mathrm{cb}}\left(r\right)}{\bar{\alpha_{\mathrm{ctx}}}\left(r\right)},$$

$$\tilde{\beta }\left(r\right)=\frac{\beta_{\mathrm{cb}}\left(r\right)}{\bar{\beta_{\mathrm{ctx}}}\left(r\right)},$$

$$\Rightarrow \tilde{\alpha }=\tilde{C}\tilde{\beta }$$

where cb denotes the cerebellum, ctx the cortex, and the bar denotes the average.

Figure 5b (bottom row) shows that the normalized net cerebellar signal $\tilde{\alpha }$ was not a monotonic function of patch radius *r*. Starting at $\tilde{\alpha }\approx 0.7\;$for individual dipoles (Figure 4), $\tilde{\alpha }$ first decreased and then increased slightly with patch size in the region 10 < *r* < 25 mm in MEG (Figure 5b, bottom left). A similar sharp initial decline in $\tilde{\alpha }$ was observed in EEG (Figure 5b, bottom right), but in the case of EEG, $\tilde{\alpha }$ decreased monotonically with patch radius, ending at ~0.4 for *r* = 30 mm. In both EEG and MEG, the initial sharp decrease in $\tilde{\alpha }$ resulted from the normalized cerebellar conservation factor falling fast for small radii (Figure 5b, middle row), that is, the cerebellar conservation factor decreasing faster than the cortical conservation factor. The normalized net cerebellar signal norm $\tilde{\alpha }$ ranged from 0.7 to 0.4 for all *r* < 30 mm, implying that the net cerebellar signal *α*_cb_ was 30–60% smaller than the net cortical signal *α*_ctx_.

Figure 5b (top row) shows the normalized absolute signal $\tilde{\beta }$ for the cerebellum which was roughly constant across patch sizes. For MEG (Figure 5b, top left), $\tilde{\beta }$ was in the 0.56–0.7 range while for EEG (Figure 5b, top right), $\tilde{\beta }$ was in the 0.71–0.74 range.

Figure 5b (middle row) displays the normalized conservation factor $\tilde{C}$. Clearly, the variation in $\tilde{\alpha }$ over patch radius *r* was mainly due to the variation of $\tilde{C}$ since $\tilde{\alpha }=\tilde{C}\tilde{\beta }$ and $\tilde{\beta }$ was roughly constant over patch sizes. Since $\tilde{C}$ and $\tilde{\beta }$ were of comparable magnitude (0.55–0.9 and 0.55–0.75, respectively), we deduced that the cancelation effect (quantified in $\tilde{C}$) and the relatively remote location of the cerebellum (quantified in $\tilde{\beta }$) equally influenced the net cerebellar signal strength $\tilde{\alpha }$, for the physiologically realistic patch sizes examined in this study.

### 3.3 Sensitivity maps

The sensitivity of MEG and EEG to neural activity varies with location and orientation of the source currents. This dependency was studied by activating individual current dipoles in the source space and calculating the Euclidean norm of the resulting signal in the M/EEG sensor space. In EEG, for each activated dipole, the reference was set to be the average of all the sensor outputs; this reference value was subtracted from the sensor data before computing the signal norm (average electrode reference). The source location was then colored according to the resulting signal norm, resulting in a sensitivity map. The strength of the current dipole was chosen to be 100 nAm, which represents activation of a patch with a radius of about 10 mm with a conservation factor of 0.3 (Figure 3).

**Figure 6:**
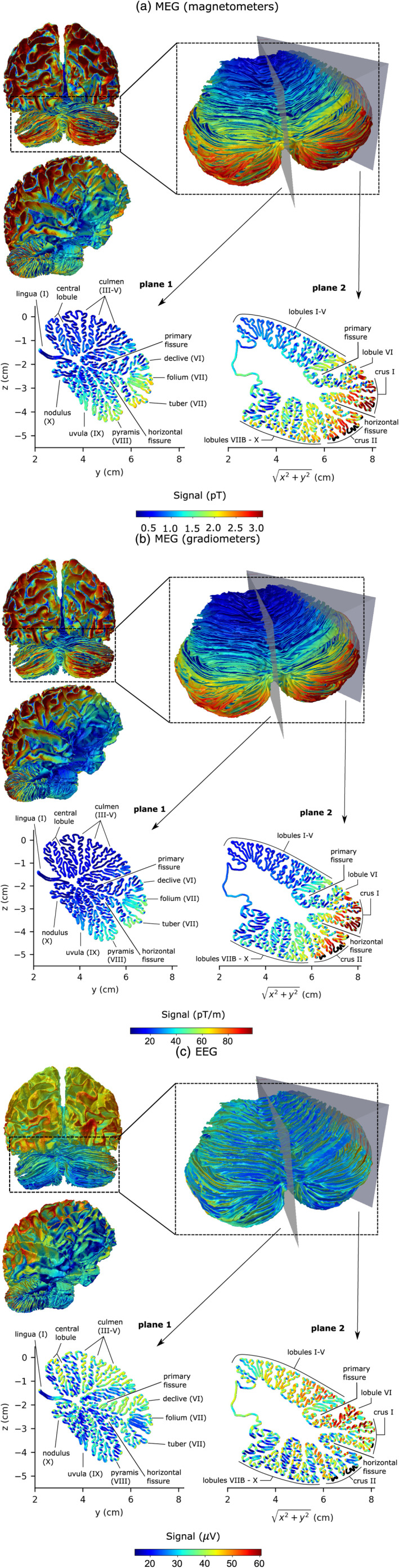
Sensitivity maps of the cerebellum and the cerebral cortex for MEG magnetometers (a), MEG gradiometers (b), and EEG (c). The color of the source corresponds to the Euclidean norm of the signal in sensor space resulting from a dipole of strength 100 nAm activated at that source point. The color scale was chosen to range from the first percentile to the 99th percentile of all cerebellar signal norms. Source points that were excluded due to the 5 mm distance limit to the inner skull boundary are black. Plane 1 is the midsagittal section of the cerebellum (vermis) and Plane 2 makes a 45° angle with the midsagittal plane in the lateral direction

Figure 6 shows detailed sensitivity maps for MEG magnetometers (Figure 6a), gradiometers (Figure 6b) and EEG (Figure 6c), respectively. The sensitivities of the magnetometer and gradiometer arrays were relatively similar although the magnetometers were more sensitive to deeper activity as compared to their sensitivity to cortical activity than the gradiometers. Both MEG and EEG were more sensitive to activity in the lateral aspects of the cerebellum than close to the midline (vermis). Both modalities were most sensitive to activity in the section between the primary fissure and the horizontal fissure, located in the superior aspect of the posterior lobe of the cerebellum. This region corresponds to lobule VI and crus I in the lateral hemispheres. EEG was also sensitive to activity in the anterior lobe of the cerebellum, while MEG was sensitive to activity in the rest of the posterior lobe, particularly crus II. The sensitivity distributions of MEG and EEG in the cerebellum were thus quite complementary. Furthermore, it is known that the sensitivity of M/EEG is greatly affected by the orientation of the source current as MEG tends to be relatively more sensitive to neural currents in the direction tangential to the head surface than in the radial direction while EEG tends to be more sensitive to radial directions. This fact becomes even more prominent in the cerebellum due to its finely convoluted cortex, as the cross‐sectional images in Figure 6 show. MEG and EEG are thus complementary not only on the larger lobular scale but also on a smaller spatial scale over the individual cerebellar sulci.

## 4 DISCUSSION

### 4.1 Significance of noninvasive recording of cerebellar electrophysiology

While the cerebellum has traditionally been viewed as a brain region mainly engaged with lower functions such as motor coordination, recent findings have shown that the human cerebellum has a far more diverse role than previously thought with an intricate topography over the cerebellar cortex (Guell, Schmahmann, Gabrieli, & Ghosh, 2018; Hoche, Guell, Sherman, Vangel, & Schmahmann, 2016; Schmahmann, 2004; Schmahmann & Sherman, 1998b; Stoodley & Schmahmann, 2009). Many theories state that the cerebellum contains internal neural models of objects to be controlled (Ebner & Pasalar, 2008; Kawato & Gomi, 1992; Miall, Weir, Wolpert, & Stein, 1993; Wolpert, Miall, & Kawato, 1998). Most theories agree that the internal models are coded by simple spikes in Purkinje cells elicited by discharging of granule cells; granule cell axons form the parallel fibers which make excitatory synaptic contact with the Purkinje neurons. These internal models are fine‐tuned by complex spikes transmitted by climbing fibers that cause plasticity in the parallel fiber‐Purkinje cell synapses by means of long‐term depression (Albus, 1971; Kitazawa, Kimura, & Yin, 1998; Marr, 1969; Medina & Lisberger, 2008). This model is strongly supported by empirical data from single cell recordings in animal models (Ito, 1982; Ito & Kano, 1982), although it has recently been challenged in gene knockout mice studies (Schonewille et al., 2011). The cerebellum has also been suggested to code the neural representation of time, which has been extensively studied via computational modeling (Ivry & Spencer, 2004).

Despite this abundance of models of cerebellar function and deep knowledge of cerebellar structure and connectivity due to the highly regular microstructure in the cerebellar cortex, cerebellar functionality has remained elusive. One major reason for this knowledge gap is the lack of empirical electrophysiology data that are not from invasive single cell recordings in animal models but from whole neuronal assemblies in healthy humans. With M/EEG, it should be possible to obtain these empirical data, but an analytical approach evaluating the feasibility of doing so has been lacking. The present work aimed to bridge this gap by assessing the cerebellar signal strength in the M/EEG data.

### 4.2 Cerebellar versus cortical M/EEG signal strength

The simulations in this article suggest that the cerebellar signal strength in both MEG and EEG is only 30–60% smaller than the cortical signal strength for activated patch areas of similar sizes (Figure 5). This finding along with the observation that the cerebellum is engaged in a wide range of tasks (Stoodley & Schmahmann, 2009), suggests that there is likely a detectable cerebellar M/EEG signal in many more experimental settings than formerly assumed. Considering the relatively sparse body of M/EEG literature that has reported cerebellar activity raises the possibility that some cerebellar signals in the M/EEG sensor space data may have been wrongly mapped to the neighboring occipital lobe. This is particularly pertinent in studies that use a surface source space consisting of just the cerebral hemispheres excluding the cerebellum, which is sometimes the case when using software like Brainstorm or MNE‐Python. For this reason, using beamformers with a volumetric source space may be a better choice for source reconstruction. A future study on cerebellar inverse modeling is warranted to investigate this further.

### 4.3 Underestimation of signal cancelation using the FreeSurfer cerebellum model

The simulations in this paper showed that using the standard FreeSurfer segmentation as the source space for cerebellar forward modeling significantly underestimated the amount of signal cancelation (Figure 3) and therefore overestimated the net cerebellar signal in sensor space. A more detailed analysis of the predictions based on the low‐resolution FreeSurfer model is presented in the Appendix (Figure A1). We recommend that future studies of the cerebellum therefore employ either a volumetric source space, in which a priori anatomical information is not fully utilized, or a surface source space made by warping the high‐resolution cerebellar cortex to align with the cerebellum of individual subjects, as was done in the present study. Ideally, one would acquire a <200 μm‐resolution MRI scan of the individual subject's cerebellum but this is not possible in vivo with current MRI technology. While further work is required to characterize intersubject variability in cerebellar structure down to the folia‐level, the method used in this article could serve as a better approximation of the true source space in healthy subjects and in disease populations with little or no structural changes.

**Figure 7:**
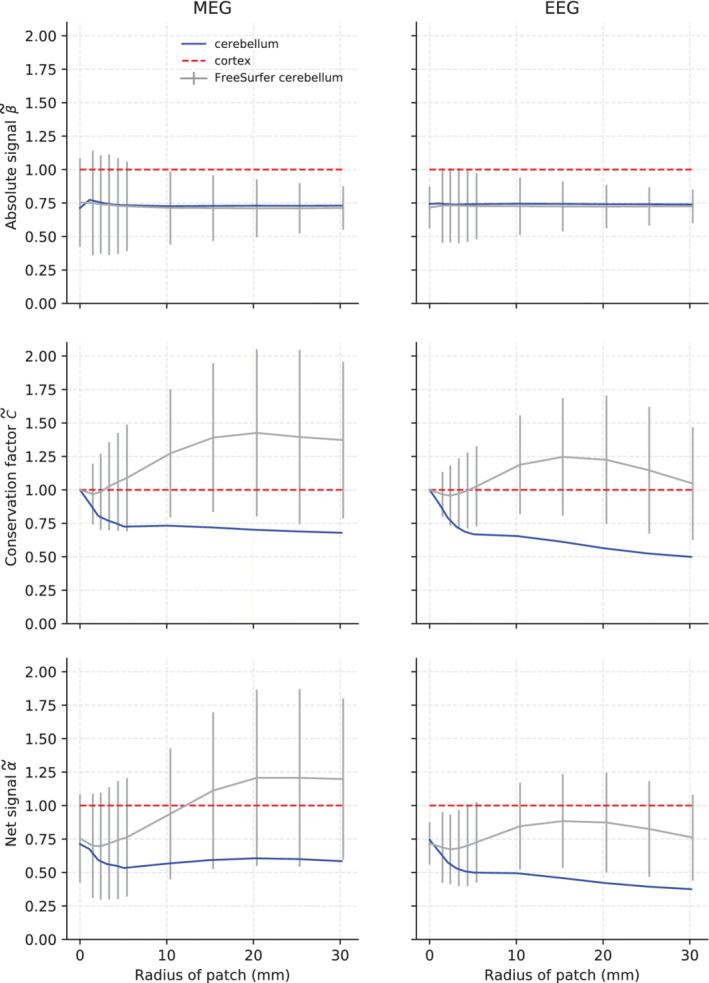
Absolute Euclidean signal norm (top row), conservation factor (middle row), and net Euclidean signal norm (bottom row) for MEG magnetometers (left column) and EEG electrodes (right column) from 200 samples of varying activated patch sizes in the low‐resolution FreeSurfer cerebellum (gray). The solid line represents the median value and the error bars plus/minus one standard deviation. The median values for the high‐resolution cerebellum model (blue) have been added for reference. All data were normalized to the mean of the cortical conservation factor or signal norm (dashed red line)

### 4.4 Designing future M/EEG cerebellar studies using the detailed sensitivity maps

The detailed sensitivity maps (Figure 6) should serve as a useful aid when designing future MEG and EEG studies aimed at detecting cerebellar signals. Due to the typically low SNR in M/EEG recordings and its high temporal resolution (Hämäläinen et al., 1993), stimuli that are repeatable and render a phase‐locked neural response are preferable, so that the M/EEG signal can be averaged and filtered in the frequency band of interest or correlated with the stimulus, thus significantly increasing SNR. Although cognitive tasks usually do not elicit neural responses that are phase‐locked in time to the stimulus, there are many stimuli that are repeatable and cause de and resynchronization of brain rhythms in specific frequency bands. A phase‐locked response can therefore be elicited by presenting the stimulus at a fixed rate, as this will result in an amplitude modulation waveform of the brain rhythm being examined. Examples of experimental paradigms that are repeatable and activate cerebellar areas that should give strong M/EEG signals include nociceptive withdrawal reflex (lateral lobule VI/crus I; Dimitrova et al., 2003), eye blink response (crus I/lobule VI; Gerwig, Kolb, & Timmann, 2007; Ramnani, Toni, Josephs, Ashburner, & Passingham, 2000), and working memory tasks (lobules VI, VIIb, VIII, crus I/II; Guell et al., 2018; Hautzel, Mottaghy, Specht, Müller, & Krause, 2009; Koelsch et al., 2009). Working memory tasks in particular should elicit strong cerebellar signals in both MEG and EEG, since their activation region is located favorably for M/EEG detection according to our sensitivity maps (Figure 6) and involve an extensive surface area, which is preferable since the M/EEG signal strength increases linearly with activated surface patch radius (Figure 5).

Reported M/EEG studies have elicited cerebellar activations with median nerve stimulation, finger tapping, and saccades. Median nerve stimulation is reported to activate the paravermal region of the superior aspect of the posterior lobe (approximately lobules VI, VII; Hashimoto et al., 2003) or the vermis around the primary fissure (approximately lobules V and VI; Tesche & Karhu, 1997); the same region activated during intermittent omission of stimulation (Tesche & Karhu, 2000). Saccades were reported to activate the flocculonodular lobe and the posterior vermis (Ioannides et al., 2005; Jousmaki et al., 1996) and finger tapping was reported to activate the ipsilateral paravermis region (likely crus I; Muthuraman et al., 2014). Auditory working memory activation was also reported by Wibral et al. (2011). These regions generally agree well with our sensitivity maps; M/EEG should have good coverage of the reported activation sites except for the flocculonodular lobe, which is very deep and close to the midbrain, with poor MEG coverage. The presence of two bilateral dipoles that are aligned and lie close to each other may still give an appreciable signal.

### 4.5 Comparison of EEG and MEG for detection of cerebellar activity

Our simulations revealed that while EEG and MEG showed comparable average cerebellar signal strengths in relation to their respective cortical signals, they had highly complementary sensitivity distributions. The well‐known difference in sensitivity to current dipole orientation between MEG and EEG is even more prominent in the cerebellum due to the highly convoluted cerebellar cortex. Unlike the cortex where sulci usually do not contain sub‐sulci, each folium in the cerebellum has multiple small sulci and gyri, making the curvature of the cerebellar cortex very high and the spatial sensitivity gradient consequently much higher in the cerebellum than in the cortex.

Although both MEG and EEG were generally mostly sensitive to activity in lobule VI and crus I in the lateral hemispheres of the cerebellum (Figure 6), there was also a difference in sensitivity on a larger spatial scale. Apart from lobule VI and crus I which are in the superior region of the posterior lobe, EEG was sensitive to activity in the anterior lobe of the cerebellum while MEG was sensitive to activity in the rest of the posterior lobe (Figure 6). This might lead one to conclude that MEG is a better tool for investigating cognitive aspects of the cerebellum occurring in the posterior lobe while EEG is better suited for studying activity related to motor functions, since these mainly occur in the anterior lobe of the cerebellum (Schmahmann, 2004; Schmahmann & Sherman, 1998b; Stoodley & Schmahmann, 2009). However, the EEG sensors near the cerebellum are plagued by myogenic contaminations from the splenius muscles and neural signals from the occipital lobe which render interpretation of the electrophysiology data challenging. A recent study by Todd et al. (2018), however, showed promising experimental results on recording cerebellar oscillations with EEG using well‐placed electrodes over the posterior fossa while using electrodes over the occipital lobe and splenius muscles as reference sensors. It is conceivable that one could use such reference sensor data in a signal subspace projection technique like cortical signal suppression (Samuelsson, Khan, Sundaram, Peled, & Hämäläinen, 2019) to suppress the occipital and myogenic contaminations in the sensor space data. All these observations taken together, it appears that the optimal measurement system for studying cerebellar electrophysiology is combined M/EEG with reference sensors over the splenius muscles controlling for myogenic contamination, much like electrodes to the left of the sternum are used today to control for myocardial contaminations in MEG.

### 4.6 Potential relevance to cerebellar TMS

Recently, transcranial magnetic stimulation (TMS) has been used to study functions of the human cerebellum because it can noninvasively modulate connectivity between the cerebellum and the primary motor cortex (Daskalakis et al., 2004; Grimaldi et al., 2014; Miall & Christensen, 2004; Minks, Kopickova, Marecek, Streitova, & Bares, 2010) as well as evoke responses in the motor cortex and affect limb movements (Fierro et al., 2007; Miall & Christensen, 2004; Oliveri, Koch, Torriero, & Caltagirone, 2005). Because there exists an electromagnetic reciprocity between MEG and TMS (Heller & van Hulsteyn, 1992), methods like the ones we developed in this article for assessing MEG sensitivity can be applied to the stimulation problem as well (van Dun, Bodranghien, Manto, & Marien, 2017).

### 4.7 Future studies

This study was devoted to a theoretical investigation of the cerebellar signals in M/EEG. This included quantifying signal cancelation, signal strength, and comparing these results to the cortical signals as well as computing sensitivity maps outlining how the signal strength varies over the cerebellar cortex for different modalities. The question of whether cerebellar activity can be reconstructed reliably in practice based on noisy M/EEG data, and what practices are suitable for this purpose, for example, choice of inverse method and source space, should be the topic of a future study. Although this study clearly showed that a full surface source space that resolves the folia was necessary for doing proper forward modeling to avoid underestimation of cancelation effects, it is entirely feasible that a volumetric source space may be sufficient for inverse modeling purposes. Although we noted here that MEG and EEG have complementary sensitivity distributions and magnetometers can see deeper into the cerebellum, different MEG and EEG sensors have different noise levels which vary across M/EEG systems, subjects, and experiments; the relative SNR could thus be different from the relative signal strengths. Future studies on inverse modeling are needed to take these differing noise levels into consideration.

## 5 CONCLUSION

Our simulations suggest that the average cerebellar signal in MEG and EEG is weaker (30–60%) than the cortical signal but of the same order‐of‐magnitude, despite higher signal cancelation. MEG and EEG were found to have highly complementary sensitivity distributions; we therefore recommend combined M/EEG measurement for studying cerebellar electrophysiology noninvasively. The high‐resolution cerebellar sensitivity maps for EEG and MEG developed in this study will likely serve as a useful tool for future design of noninvasive cerebellar electrophysiology studies. Our results suggest that neural activity in the cerebellum should be clearly detectable by current M/EEG systems.

## CONFLICT OF INTEREST

The authors declare no potential conflict of interest.

## Data Availability

The data that support the findings of this study are available from the corresponding author upon reasonable request.

## APPENDIX 1

Figure A1 shows the net signal, absolute signal, and conservation factor using the high‐resolution cerebellar model used in this study and the low‐resolution FreeSurfer reconstruction that is based on conventional MRI data, normalized to the cortical values, the same way as was done in Figure 5b. In both MEG and EEG, the low‐resolution FreeSurfer model predicted a higher cerebellar conservation factor than that of the cortex for most patch sizes, which resulted in a gross over‐estimation of the net signal.
